# Identification and Characterization of Three New Cytochrome P450 Genes and the Use of RNA Interference to Evaluate Their Roles in Antioxidant Defense in *Apis cerana cerana* Fabricius

**DOI:** 10.3389/fphys.2018.01608

**Published:** 2018-11-15

**Authors:** Weixing Zhang, Wenfeng Chen, Zhenfang Li, Lanting Ma, Jing Yu, Hongfang Wang, Zhenguo Liu, Baohua Xu

**Affiliations:** College of Animal Science and Technology, Shandong Agricultural University, Tai’an, China

**Keywords:** *Apis cerana cerana* Fabricius, cytochrome P450s, RNA interference, abiotic stresses, antioxidant enzyme

## Abstract

Cytochrome P450s play critical roles in maintaining redox homeostasis and protecting organisms from the accumulation of toxic reactive oxygen species (ROS). The biochemical functions of the P450 family have essentially been associated with the metabolism of xenobiotics. Here, we sequenced and characterized three P450 genes, *AccCYP314A1, AccCYP4AZ1*, and *AccCYP6AS5*, from *Apis cerana cerana* Fabricius; these genes play a critical role in maintaining biodiversity. Quantitative PCR (qPCR) analysis indicated that the three genes were all predominantly expressed in the epidermis (EP), followed by the brain (BR) and midgut (MG). In addition, the highest expression levels were detected in the dark-eyed pupae and adult stages. The three genes were induced by temperature (4°C and 44°C), heavy metals (CdCl_2_ and HgCl_2_), pesticides (DDV, deltamethrin, and paraquat) and UV treatments. Furthermore, Western blot analysis indicated that the protein expression levels could be induced by some abiotic stressors, a result that complements the qPCR results. We analyzed the silencing of these three genes and found that silencing these genes enhanced the enzymatic activities of peroxidase (POD) and catalase (CAT). Additionally, we investigated the expression of other antioxidant genes and found that some were upregulated, while others were downregulated, suggesting that the upregulated genes may be involved in compensating for the silencing of *AccCYP314A1, AccCYP4AZ1*, and *AccCYP6AS5*. Our findings suggest that *AccCYP314A1, AccCYP4AZ1*, and *AccCYP6AS5* may play very significant roles in the antioxidant defense against damage caused by ROS.

## Introduction

As social insects and pollinators of flowering plants, Chinese honeybees (*Apis cerana cerana* Fabricius, *A*. *c. cerana*) play an important role in the balance between agricultural economic development and regional ecology. Compared with *Apis mellifera* (*A. mellifera*), *A.* *c. cerana* has acquired some incomparable advantages through the history of evolution. *A.* *c. cerana* has a strong resistance to mites, an acute sense of smell, and can forage more widely of pollens, which are irreplaceable (Oldroyd and Wongsiri, 2006). Nevertheless, the population of *A.* *c. cerana* has been reported to have severely declined, which has been referred to as colony collapse disorder (CCD), likely due to various abiotic stresses that exist in the environment, such as extreme changes in temperature and widely used insecticides that may generate reactive oxygen species (ROS). Therefore, understanding the antioxidant system and its molecular mechanisms of defense against ROS damage has become a primary focus of research.

Excessive ROS can lead to lipid peroxidation and protein damage, which cause cell apoptosis and the loss of enzymatic activity, respectively (Green and Reed, 1998; Stadtman and Levine, 2003). Sperm storage was also affected by ROS in *A. mellifera* (Collins et al., 2004). Organisms protect themselves through a range of antioxidant enzymes, such as catalase (CAT), glutathione-S-transferases (GSTs), superoxide dismutase (SOD), peroxidases (POD), glutathione peroxidase (GPX), and cytochrome P450s (P450s) (Dubovskiy et al., 2008; Matteis et al., 2012).

P450s, a supergene family, are widely distributed in almost all living organisms and play crucial roles in insect biology and physiology (Feyereisen, 1999). P450s are classified into different clades on the basis of their evolutionary relationships (Claudianos et al., 2006; Moktali et al., 2012). In insects, P450s belong to four clades: the mitochondrial P450s (Mit P450s), CYP2 clades, CYP3 clades (including CYP6 and CYP9), and CYP4 clades. The Mit P450s and CYP2 clades have been implicated in essential roles in hormone biosynthesis (Gilbert, 2004; Semak et al., 2008; Sangar et al., 2010). Meanwhile, the CYP3 and CYP4 clades are largely responsible for abiotic stresses, such as pesticide metabolism, detoxifying functions, and chemical communication (Yu et al., 1984; Claudianos et al., 2006; Musasia et al., 2013).

Previous studies concerning the roles of CYP3 P450s have focused on their functions in the environmental response and detoxification in insects. In the pyrethroid-resistant *Anopheles gambiae, CYP6Z1* (belonging to the CYP3 clade) is overexpressed (Nikou et al., 2003). Similarly, in *Helicoverpa zea, CYP321A1*, a member of the CYP3 clade, can metabolize cypermethrin (Sasabe et al., 2004). Meanwhile, CYP3A enzymes play a crucial role in preventing the bioaccumulation of xenobiotics substances (drugs, environmental pollutants, and fungal products) that enter the body and metabolize endogenous substrates, such as sex hormones, in steroidogenic tissues (Celander et al., 2000; Danielson, 2002; Hegelund and Celander, 2003).

In insects, the CYP4 clade, a highly diversified group of enzymes, are involved in both pesticide metabolism and chemical communication (Scott et al., 1994; Claudianos et al., 2006; Kirischian and Wilson, 2012). For example, some CYP4 clade genes are over expressed in several pesticide-resistant insects (*Culex pipiens* and *Diabrotica virgifera virgifera*), and *CYP4G8* is over expressed in pyrethroid-resistant strains of *Helicoverpa armigera* (Pittendrigh et al., 1997; Scharf et al., 2001; Shen et al., 2003). Moreover, the expression of CYP4G11 was significantly induced by external factors such as temperature challenges, UV radiation, insecticide treatment, and the expression patterns under oxidative stress, suggesting that CYP4G11 may be involved in protecting honeybees from oxidative injury (Shi et al., 2013). These studies demonstrate that the CYP3 and CYP4 clades play key roles in protecting organisms from ROS damage.

Although the functions of the P450s have been investigated in other species, there is limited knowledge regarding the P450s in honeybees, particularly in *A. c. cerana*. The above discussion demonstrates that the P450s play key roles in the response to oxidative stress in most species, and we predict that the P450s may also be involved in antioxidant defense in *A. c. cerana*. To gain insight into the roles of the P450s, we isolated and characterized three genes (*AccCYP314A1, AccCYP4AZ1*, and *AccCYP6AS5*) from *A. c. cerana* and analyzed the expression patterns of these genes in different tissues and different developmental stages. We also investigated the transcripts of these three genes when exposed to various oxidative stresses, including different temperatures (4 and 44°C), heavy metals (HgCl_2_ and CdCl_2_), pesticides (DDV, deltamethrin, and paraquat), and ultraviolet light (UV). Moreover, we used RNAi technology to knockdown *AccCYP314A1, AccCYP4AZ1*, and *AccCYP6AS5*. We also investigated the activity of two antioxidant enzymes (POD and CAT) and examined the expression levels of other antioxidant genes. A very broad conclusion can be drawn from our results, with some considerable degree of certainty: *AccCYP314A1, AccCYP4AZ1*, and *AccCYP6AS5* might play crucial roles in the response to oxidative stress in *A. c. cerana*.

## Materials and Methods

### Laboratory Feeding of Honeybees and Experimental Design

Animal housing facilities and handling protocols were approved by the Animal Welfare and Health Committee of Shandong Agricultural University. Honeybees were collected from the experimental apiary of Shandong Agricultural University (Tai’an, China). Honeybees of different development stages, including larvae (L1–L5), pupae [pre-pupal phase (Pp), white-eyed pupae (Pw), brown-eyed pupae (Pb), and dark-eyed, dark pigmented thorax pupae (Pbd)] (de F Michelette and Soares, 1993), and newly emerged worker bees (less than 12 h of age), were obtained from five healthy hives. Newly emerged workers were collected at 15 days after being labeled with paint. The brain (BR), midgut (MG), muscle (MS), and epidermis (EP) of 15-day post-emergence adults (*n* = 90; 15/group) were dissected on ice, frozen immediately in liquid nitrogen, and stored at -80°C until ready for use. Adult bees (post-emergence age: 15 days) were collected in wooden cages (dimensions of 10 cm × 7 cm × 8 cm), which were maintained in an incubator (33 ± 1°C, 60 ± 10% relative humidity, darkness). Cages were divided into nine groups (*n* = 60/group). Groups 1 and 2 were fed with a sucrose solution containing HgCl_2_ and CdCl_2_, respectively. Group 3 was subjected to UV light. Groups 4 and 5 were treated with different temperatures. In addition, groups 6–8 were treated with one of three different pesticides (DDV, deltamethrin, and paraquat). Group 9 served as the negative control. The abiotic stress conditions for each experimental group are shown in Supplementary Table S1. The final effective concentrations of the pesticides were determined according to the manufacturer’s instructions. All bee samples were immediately frozen in liquid nitrogen at the appropriate time points and were stored at -80°C until ready for use. All experiments were performed with at least six biological replicates.

### Primers and PCR Amplification Conditions

All sequences of the PCR primers and the amplification conditions are listed in Tables 1, 2, respectively. In this study, all primers used for quantitative PCR (qPCR) were designed based on the principle of quantitative primer design. The *E* value and *R*^2^ value of each qPCR primer pair are listed in Table 1. All primer pairs were synthesized by the Sangon Biotechnological Company (Shanghai, China).

**Table 1:** Primers for ORF subcloning, qPCR analysis, and dsRNA primers sequence of *AccCYP314A1, AccCYP4AZ1*, and *AccCYP6AS5* genes.

Gene	Amplification	Primer sequence (5′–3′)	Product size (bp)	Coefficient (*R*^2^)
*CYP314A1*	ORF amplication	F: CGAGATGTTACTCTCGAGTG	1,506	
		R: AGATGAACGATCTCAAGAAC		
	qPCR	F: CTTCGGGTAGCTCTCACGTC	125	0.998
		R: ACTTTGTATCCGACCCGTTG		
	dsRNA	F: TAATACGACTCACTATAGGGCGAATATTAATTTCATGCCTCAG	475	
		R: TAATACGACTCACTATAGGGCGAATACTACTGCGACTAGC		
*CYP4AZ1*	ORF amplication	F: ATGATTTCTGCAATATTGTTCTTTA	1,548	
		R: CATGATCAACATATCTTCTTAACAGG		
	qPCR	F: TTGGCCGAATCCAAATAAGT	123	0.987
		R: GCAAATCGTTGTCCAATGC		
	dsRNA	F: TAATACGACTCACTATAGGGCGAATGATTTCTGCAA	526	
		R: TAATACGACTCACTATAGGGCGATTCACTCTCTAATG		
*CYP6AS5*	ORF amplication	F: ATGGCGAGCAGTTTCGAA	1,500	
		R: TCATATTTTTGTTATTTTCAAATATATTC		
	qPCR	F: AATGGGCAGAGAAGTGTTCG	127	0.998
		R: AAAGAATGGTGCGAATGTCC		
	dsRNA	F: TAATACGACTCACTATAGGGCGAATGGCGAGCAGTTT	507	
		R: TAATACGACTCACTATAGGGCGAGCATTCGATTATGAG		
GFP	dsGFP	F: TAATACGACTCACTATAGGGCGAAGTGGAGAGGGTGAAGGTGA	546	
		R: TAATACGACTCACTATAGGGCGAGGTAAAAGGACAGGGCCATC		
*AccTrx1*	qPCR	F: GGTTTGAGAATTATACGCACTGC	124	0.989
		R: GAGTAAGCATGCGACAAGGAT		
*AccsHSP22.6*	qPCR	F: CGATGAGCACGGTTGGATTTCAC	127	0.999
		R: GGTTCTGCTGCTGTTTGGGTG		
*AccGSTO1*	qPCR	F: CATTCTTTCATGGTAATTCTCCTGGC	123	0.999
		R: TTAATCAGTAATCAAATCATATTGTGG		
*β-acting*	qPCR	F: TTATATGCCAACACTGTCCTTT	126	0.998
		R: AGAATTGATCCACCAATCCA		


*Underlined sequences in primers for dsRNA production are added adaptors containing T7 polymerase promoters.*

**Table 2:** PCR amplification conditions.

Primer pairs	Amplification conditions
AccCYP314A1-F/R	10 min at 94°C, 40 s at 94°C, 40 s at 49°C, 60 s at 72°C for 35 cycles, 10 min at 72°C
AccCYP4AZ1-F/R	10 min at 94°C, 40 s at 94°C, 40 s at 55°C, 70 s at 72°C for 35 cycles, 10 min at 72°C
AccCYP6AS5-F/R	10 min at 94°C, 40 s at 94°C, 40 s at 54°C, 70 s at 72°C for 35 cycles, 10 min at 72°C
^∗^AccCYP314A1-F/R	10 min at 94°C, 40 s at 94°C, 40 s at 56°C, 40 s at 72°C for 35 cycles, 10 min at 72°C
^∗^AccCYP4AZ1-F/R	10 min at 94°C, 40 s at 94°C, 40 s at 57°C, 40 s at 72°C for 35 cycles, 10 min at 72°C
^∗^AccCYP6AS5-F/R	10 min at 94°C, 40 s at 94°C, 40 s at 55°C, 40 s at 72°C for 35 cycles, 10 min at 72°C
^∗^GFP-F/R	10 min at 94°C, 40 s at 94°C, 40 s at 58°C, 40 s at 72°C for 35 cycles, 10 min at 72°C


*^∗^Primers for dsRNA productions.*

### Total RNA Isolation, cDNA Synthesis, and DNA Isolation

Total RNA was extracted from samples using the E.Z.N.A. Total RNA Kit II (OMEGA, United States), according to the manufacturer’s instructions. The purity and quality of the RNA were controlled by using a spectrophotometer and were estimated by electrophoresis. The PrimeScript RT reagent Kit with gDNA Eraser (TaKaRa, Japan) was used to generate first-strand cDNA, according to the manufacturer’s instructions.

### Cloning the cDNA Sequences of *AccCYP314A1, AccCYP4AZ1*, and *AccCYP6AS5*

To obtain the open reading frame (ORF) sequences of *AccCYP314A1, AccCYP4AZ1*, and *AccCYP6AS5*, special primers (Table 1) were designed and synthesized based on the conserved regions of the genes from *A. mellifera, Apis florea*, and *Apis dorsata*. The cDNA of newly emerged workers was used to clone the ORFs of these three genes. The PCR reaction used 1 μL cDNA as a template, 12.5 μL 2× Es Taq MasterMix (Dye) (CWBIO, Beijing, China), 1 μL forward primer, 1 μL reverse primer, and 9.5 μL ddH_2_O in a 25-μl volume with the following cycling conditions: initial denaturation program (94°C for 10 min), followed by 35 cycles of 94°C for 40 s, N°C for 40 s, 72°C for *M* s (*N*: the advisable annealing temperature of the primers; *M*: determined by the length of the gene sequences), and 72°C for 10 min. All PCR products were separated by electrophoresis and purified using a Gel Extraction Kit (Solarbio, China), cloned into pEASY-T1 vectors (TransGen, China), and transformed into competent *Escherichia coli* DH5 α (*E*. *coli* DH5 α) cells for sequencing (Sangon, China).

### Bioinformatics Analysis and Phylogenetic Tree Construction

DNAman version 5.2.2 (Lynnon Biosoft, Canada) was used to search for the ORFs of the three genes and to predict the theoretical isoelectric points (PIs) and molecular weights (MWs) of the protein products. Conserved domains in the three P450 genes were detected using bioinformatics tools available at the NCBI server.^1^ Phylogenetic analyses were conducted using Molecular Evolutionary Genetics Analysis 7 software (MEGA version 7), using the neighbor-joining method (Saitou and Nei, 1987). The bootstrap consensus tree inferred from 500 replicates is taken to represent the evolutionary history of the analyzed taxa (Felsenstein, 1985). Branches corresponding to partitions reproduced in less than 50% of the bootstrap replicates are collapsed. The evolutionary distances were computed using the Poisson correction method (Felsenstein, 1985) and are expressed as the number of amino acid substitutions per site. All positions containing alignment gaps and missing data were eliminated only in pairwise sequence comparisons (pairwise deletion option). There was a total of 507 positions in the final dataset.

### Real-Time Relative Quantitative PCR

Real-time relative qPCR was used to analyze the mRNA levels of the three P450 genes using the 7500 Real-time System (ABI, United States) and ABI SDS 1.4 for the 7500 system (ABI, United States). The *A. c. cerana β-actin* gene (GenBank accession no. XM_017065464) was used as a reference gene. The levels of the target genes were compared among the groups of interest. qPCR reactions were performed in a final volume of 20 μL:7 μL sterile deionized water, 1 μL each primer (10 μmol/L), 10 μL Taq DNA polymerase (5 U/μL, TaKaRa), and 1 μL DNA template (50 ng/μL). All qPCR amplifications were performed using the following conditions: (1) 30 s at 95°C for pre-denaturation; (2) 40 cycles of amplification (5 s at 95°C for denaturation, 35 s at 60°C for annealing and extension). Three technical replicates were performed for each experiment.

### Antibody Production

Custom-made polyclonal antibodies were used. The epitopes were predicted using the GenScript OptimumAntigen design tool, and the peptide antigen sequences for *AccCYP314A1, AccCYP4AZ1*, and *AccCYP6AS5* were PFGAGRRICPGK, EAHRNNKIDDEGIRE, and PNPDSFDPERFDQDAMAS, respectively. The peptide antigens were then synthesized (Sangon, China). After the coupling reaction and mixing with complete adjuvant, the coupled antigen was used for injection. Next, the coupled antigen was mixed with incomplete adjuvant (Sigma, United States) and injected into white mice (Taibang, China), which were 6 weeks old and specific pathogen-free, four times at 4-week intervals. Subsequently, blood was collected by eyeball puncture, incubated at 37°C for 1 h, and centrifuged at 3,000 × g for 10 min. Finally, the anti-serum was prepared. The collected antibody was hybridized to a blot containing the overexpressed proteins to detect the specificity of the anti-serum.

### Western Blotting

The honeybee whole bodies were lysed in RIPA buffer (pH 7.5). The lysate was centrifuged at 10,000 × g for 15 min at 4°C. The supernatant was then collected, and the protein concentration was determined using the BCA Protein Assay Kit (Beyotime, China). The extracted protein was separated on a 10% SDS-PAGE gel and transferred onto PVDF membranes (Millipore, Bedford, MA, United States). The membrane was blocked with QuickBlock^TM^ Western buffer (Beyotime, China) for 1.5 h to reduce non-specific binding. Next, the blot was incubated with the primary antibody overnight at 4°C. After washing, the blot was incubated with AP-labeled goat anti-mouse IgG (H + L) secondary antibody (Beyotime, China) for 4 h at 4°C. Finally, the signal was detected using an enhanced BeyECL Plus kit (Beyotime, China) and visualized in Fusion Fx by Vilber Lourmat. The optical density of each band was quantified using Fusion Capt Advance Fx7 software (Beijing Oriental Science, China), using tubulin as an internal control. The antibodies used included anti-AccCYP314A1 (1:100), anti-AccCYP4AZ1 (1:100), anti-AccCYP6AS5 (1:100), and anti-tubulin (1:1,000).

### RNA Interference

To synthesize dsRNA for *AccCYP314A1, AccCYP4AZ1*, and *AccCYP6AS5*, gene-specific primers (listed in Table 1), with a T7 polymerase promoter sequence at their 5′ ends, were used to amplify the target sequences of the three genes by PCR. The PCR amplification conditions are shown in Table 2. The PCR products were purified using the Gel Extraction Kit (Solarbio, China). Next, the dsRNAs for the three P450 genes were synthesized using Ribo^MAX^ T7 large-scale RNA production systems (Promega, United States), according to the manufacturer’s instructions. To remove the DNA template, the synthesized dsRNA was digested using DNase I, precipitated with absolute ethyl alcohol, and then redissolved in RNase-free water. dsRNA of the green fluorescent protein gene (*GFP*) (GFP control; GenBank accession no. U87974) was also synthesized.

Newly emerged workers, divided randomly into six groups (*n* = 50/group), were used for RNA interference (RNAi) experiments. Four groups were injected with 0.5 μL (9 μg) of either ds*AccCYP314A1*, ds*AccCYP4AZ1*, ds*AccCYP6AS5* or ds*GFP*, performed as previously described by Amdam et al. (2003) (Hossain et al., 2008). The fifth group was injected with 0.5 μL of sterile water (H_2_O control), and the sixth group was the negative control group without treatment. Honeybees were maintained in an incubator (60% relative humidity at 34°C) under a 24-h darkness regimen. Healthy workers were subsequently sampled each day and flash frozen in liquid nitrogen and stored at -80°C until ready for use. qPCR was performed to detect *AccsHsp22.6* (GenBank accession no. KF150016), *AccGSTO1* (GenBank accession no. KF496073), and *AccTrx1* (GenBank accession no. JX844652) expression profiles when *AccCYP314A1, AccCYP4AZ1*, and *AccCYP6AS5* were knocked down. Six independent biological replicates were performed in each experiment.

### Enzymatic Activities of RNAi-Mediated Silencing Samples of *AccCYP314A1, AccCYP4AZ1*, and *AccCYP6AS5*

Total proteins were extracted from adults injected with ds*AccCYP314A1*, ds*AccCYP4AZ1*, ds*AccCYP6AS5* or ds*GFP* at 2 and 3 days post injection. The total proteins were quantified using the BCA Protein Assay Kit (Beyotime, China). Next, a CAT test kit (Jiancheng, China) was used to assay CAT capacity. In addition, a POD assay kit (Jiancheng, China) was used to assay the POD capacity, according to the manufacturer’s protocols.

### Assessment of Survival Rate

Honeybees were randomly assigned to six experimental groups with three cages per group (*n* = 3; 1,080 bees in total). Groups I and II were injected with 0.5 μL (9 μg) dsRNA-*AccCYP314A1*; groups III and IV were injected with 0.5 μL (9 μg) dsRNA-*AccCYP4AZ*1; groups V and VI were injected with 0.5 μL (9 μg) dsRNA-*AccCYP6AS5*. Groups I, III, and V were fed with a 50% sucrose diet (control group, CK); groups II, IV, and VI were fed diets containing DDV, deltamethrin, and paraquat, respectively. The pesticides conditions for each experimental group are shown in Supplementary Table S1. The number of dead bees in the treatment groups were carefully recorded, and the dead were discarded (Malone and Burgess, 2001).

### Statistical Analysis

The significant differences were determined by a Tukey’s honestly significant difference test (Tukey’s HSD) using the Statistical Analysis System version 9.1 software program (SAS, United States). Equivalence of variance among groups was evaluated using the Levene’s Test for homogeneity of variance. One-way ANOVA was used for the analyses. The data are presented as the mean + SEM (*n* = 6). For the same column, values with different small letter superscripts indicate a significant difference (*P* < 0.05), while those with the same or no letter superscripts indicate no significant difference (*P* > 0.05).

## Results

### Isolation of the Sequences for *AccCYP314A1, AccCYP4AZ1*, and *AccCYP6AS5* and Analysis

The ORF sequence of *AccCYP314A1* (GenBank accession no. MH748719) was 1,554 bp. The ORF of *AccCYP314A1* encodes a polypeptide of 517 amino acids, with a calculated molecular mass of 58.6 kDa and a theoretical PI of 8.48. The length of the *AccCYP4AZ1* ORF sequence (GenBank accession no. MH748718) was 1,548 bp, encoding a polypeptide of 515 amino acids, with a calculated molecular mass of 61 kDa and a theoretical PI of 9.07. The *AccCYP6AS5* ORF (GenBank accession no. MH748720) was 1,500 bp long. The ORF encoded a polypeptide of 499 amino acids, with a predicted molecular mass of 58.57 kDa and an pI of 8.57.

As shown in Supplementary Figures S1–S3, multiple sequence alignments also revealed that the deduced amino acid sequences of each P450 (AccCYP314A1, AccCYP4AZ1, and AccCYP6AS5) have a high similarity with those of other insect genes. The deduced amino acid sequence of AccCYP314A1 was closely related to those of *A. mellifera* 314A1 (GenBank accession no. XP_006610075, 96.36% protein sequence identity), *A. florea* 314A1 (GenBank accession no. XP_012348791, 94.88% protein sequence identity), and *A. dorsata* 314A1 (GenBank accession no. XP_006619840, 94.5% protein sequence identity). The deduced amino acid sequence of AccCYP4AZ1 was closely related to those of *A. mellifera* 4AZ1 (GenBank accession no. XP_006610075, 100% protein sequence identity), *A. florea* 4AZ1 (GenBank accession no. XP_012348791, 82.14% protein sequence identity), and *A. dorsata* 4AZ1 (GenBank accession no. XP_006619840, 93.98% protein sequence identity). The deduced amino acid sequence of AccCYP6AS5 had 99.4 and 74.75% similarity with *A. mellifera* 6AS5 (GenBank accession no. NP_001035324), and *Habropoda laboriosa* 6a2-like (GenBank accession no. XP_017796836), respectively. The characteristic active-site motifs Helix-K (EXXR) (Grahamlorence et al., 1995) and Helix-C (WXXXR) (Nelson, 1998) of AccCYP314A1, AccCYP4AZ1, and AccCYP6AS5 were highly conserved among P450 genes (Supplementary Figures S1–S3). In addition, the predicted AccCYP314A1, AccCYP4AZ1, and AccCYP6AS5 proteins share several characteristics with other members of the P450 supergene family, such as the amino acid residues of FXXGXRXCXG in the haem-binding domain (Kasai and Scott, 2000) (Supplementary Figures S1–S3). As shown in the above results, we named the three P450 genes *AccCYP314A1, AccCYP4AZ1*, and *AccCYP6AS5*.

**FIGURE 1:**
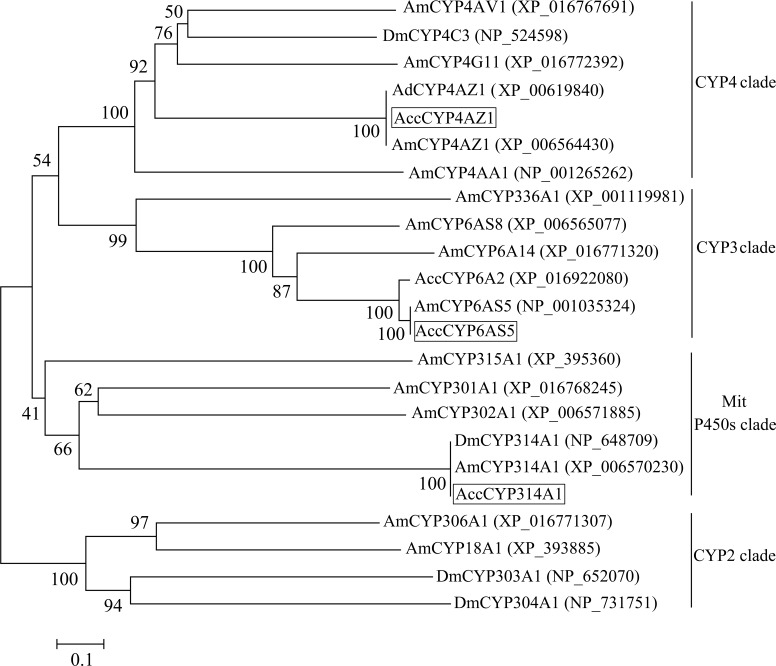
Phylogenetic analysis of *AccCYP314A1, AccCYP4AZ1*, and *AccCYP6AS5* with other known P450s genes sequences. *AccCYP314A1, AccCYP4AZ1*, and *AccCYP6AS5* are marked with the boxes.

Multiple sequence alignment revealed the nucleotide identity (54.84%) among the three P450 genes (Supplementary Figure S4). A phylogenetic tree was also built to investigate the evolutionary relationships among AccCYP314A1, AccCYP4AZ1, AccCYP6AS5 and their homologs in insects. As shown in Figure 1, the phylogenetic tree revealed that AccCYP314A1, AccCYP4AZ1, and AccCYP6AS5 belong to the mitochondrial P450 clade, the CYP4 clade and the CYP3 clade, respectively.

### Tissue-Specific Distribution and Developmental Expression of *AccCYP314A1, AccCYP4AZ1*, and *AccCYP6AS5*

**FIGURE 2:**
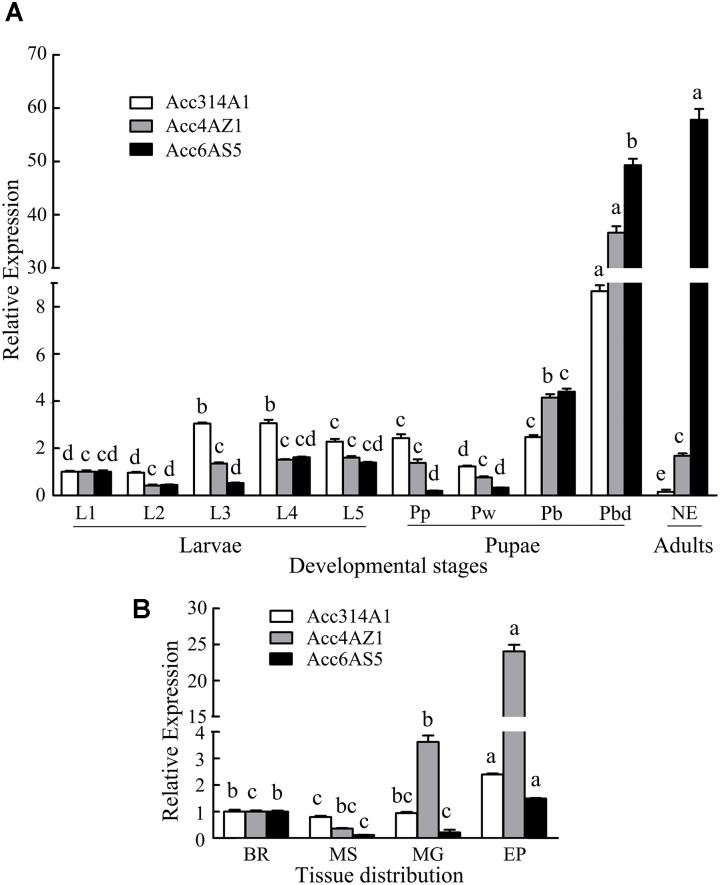
*AccCYP314A1, AccCYP4AZ1*, and *AccCYP6AS5* expression profiles in various tissues and different developmental stages. **(A)** The three genes expression levels at diverse developmental stages including 1- to 5-instar larvae (L1–L5), pre-pupal phase (Pp), white-eyed pupae (Pw), brown-eyed pupae (Pb), and dark-eyed, dark pigmented thorax pupae (Pbd), emergence worker (NE). **(B)** *AccCYP314A1, AccCYP4AZ1*, and *AccCYP6AS5* transcript levels in the brain (BR), muscle (MS), midgut (MG), and epidermis (EP). The *β-actin* gene is shown for comparison. Vertical bars and letters above vertical bars represent the mean + SEM of six different samples and significant differences (*P* < 0.05), respectively.

To investigate the expression patterns of *AccCYP314A1, AccCYP4AZ1*, and *AccCYP6AS5* in various tissues and in different developmental stages of *A. c. cerana*, qPCR was performed using total RNA extracted from various tissues from larvae, pupae, and adults. The three P450 genes were expressed differently among various tissues (Figure 2B). Specifically, *Acc314A1* was expressed predominantly in the EP, followed by the BR, and little expression was detected in MS. The relative expression level of *AccCYP4AZ1* showed the highest level in the EP, followed by the MG. There were also low levels of expression of this gene in the BR, and very little expression in the MS. *Acc6AS5* showed higher levels of expression in the EP than in any other tissues.

Developmental stage-specific expression patterns of the three P450 genes, including in first- to fifth-instar nymphs (L1–L5), pupae (Pp, Pw, Pb, and Pd), and adults, were also analyzed by qPCR. The expression pattern of the three genes were different among all the developmental stages of *A. c. cerana* (Figure 2A). Specifically, *AccCYP314A1* was predominantly expressed in Pd, followed by the third day instar larvae and the fourth day instar larvae; in adults, very little was observed. The relative expression levels of *AccCYP4AZ1* were the highest in Pd and showed the lowest expression levels in Pb. *AccCYP6AS5* was expressed at a relatively high level in adults, followed by Pd and Pb. *AccCYP4AZ1* and *AccCYP6AS5* were expressed in larvae at lower levels than pupae and NE, while *AccCYP314A1* showed the lowest in NE.

### Expression Pattern of *AccCYP314A1, AccCYP4AZ1*, and *AccCYP6AS5* Under Different Oxidative Stresses

**FIGURE 3:**
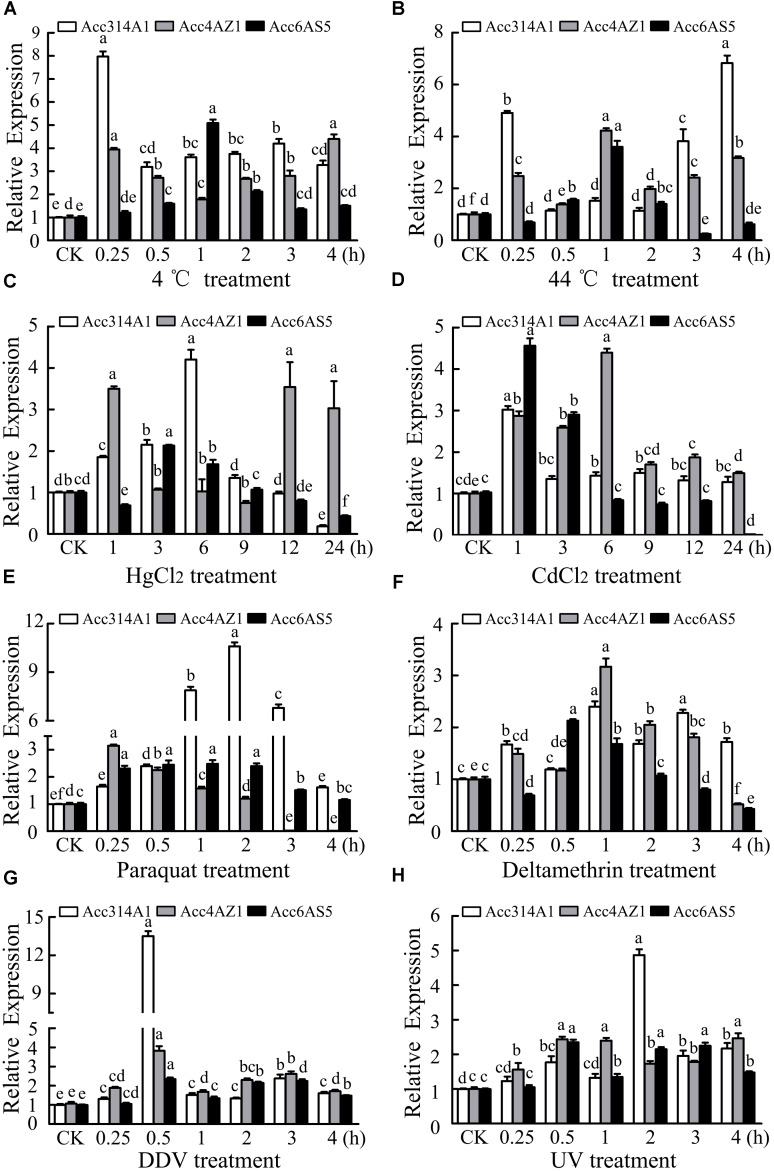
qPCR analysis of *AccCYP314A1, AccCYP4AZ1*, and *AccCYP6AS5* expression levels after treatment with various abiotic stresses. Total mRNA was extracted from adult bees (15 days post emergence) treated at the indicated times with **(A)** 4°C, **(B)** 44°C, **(C)** HgCl_2_, **(D)** CdCl_2_, **(E)** DDV, **(F)** paraquat, **(G)** deltamethrin, and **(H)** UV and qPCR was then performed. Each value is given as the mean + SEM. Different letters above the bars indicate significant differences (*P* < 0.05).

Fifteen-day post-emergence workers were exposed to 4°C, 44°C, HgCl_2_, CdCl_2_, UV light, and pesticides (DDV, paraquat, and deltamethyrin). The relative expression levels were normalized to those of the control workers (CK). As shown in Figure 3A, under 4 °C treatment, *AccCYP314A1, AccCYP4AZ1*, and *AccCYP6AS5* were all induced and reached their highest levels at 3, 4, and 1 h, respectively. *Acc314A1* was obviously induced, while *AccCYP4AZ1*, and *AccCYP6AS5* were hardly induced, when exposed to 44°C (Figure 3B). Under CdCl_2_ treatment, the transcript levels of *AccCYP314A1, AccCYP4AZ1*, and *AccCYP6AS5* were all upregulated and reached the maximum levels at 1, 6, and 1 h, respectively (Figure 3C). Under HgCl_2_ stress (Figure 3D), *AccCYP314A1, AccCYP4AZ1*, and *AccCYP6AS5* were all upregulated and reached their highest transcripts at 6, 1, and 3 h, respectively. As shown in Figure 3E, *AccCYP314A1, AccCYP4AZ1*, and *AccCYP6AS5* were upregulated when exposed to DDV and reached their peaks at 0.5 h. Under deltamethyrin and paraquat treatments, *AccCYP314A1, AccCYP4AZ1*, and *AccCYP6AS5* were all induced (Figures 3F,G). As shown in Figure 3G, *AccCYP314A1*, and *AccCYP6AS5* reached their peaks at 2.0 h, whereas *AccCYP4AZ1* reached its peak at 0.25 h. As shown in Figure 3H, the levels *AccCYP314A1, AccCYP4AZ1*, and *AccCYP6AS5* were upregulated after treatment with UV and reached maximums at 2, 0.5, and 0.5 h, respectively.

### Western Blot Analysis

**FIGURE 4:**
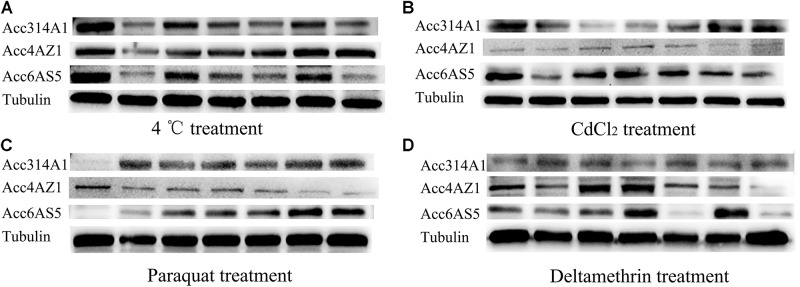
Western blot analysis of AccCYP314A1, AccCYP4AZ1, and AccCYP6AS5 changes after 4°C **(A)**, CdCl_2_ **(B)**, paraquat **(C)**, and deltamethrin **(D)** treatments.

The above qPCR results showed that the *AccCYP314A1, AccCYP4AZ1*, and *AccCYP6AS5* genes were induced in *A. c. cerana* in response to various types of oxidative stress. The entire Western blot using the three specific antibodies against the AccCYP314A1, AccCYP4AZ1, and AccCYP6AS5 proteins is shown in Supplementary Figure S5. Western blot was used to analyze the changes in AccCYP314A1, AccCYP4AZ1, and AccCYP6AS5 protein levels after 4 °C (A), CdCl_2_ (B), paraquat (C), and deltamethrin (D) treatments (Figure 4). In our Western blot results, the protein expression levels of AccCYP314A1, AccCYP4AZ1, and AccCYP6AS5 are basically consistent with the qPCR results (Figure 4), although there were small differences in the time points and degree of expression.

### Knockdown of *AccCYP314A1, AccCYP4AZ1*, and *AccCYP6AS5* and the Expression Profiles of Other Antioxidant Genes

**FIGURE 5:**
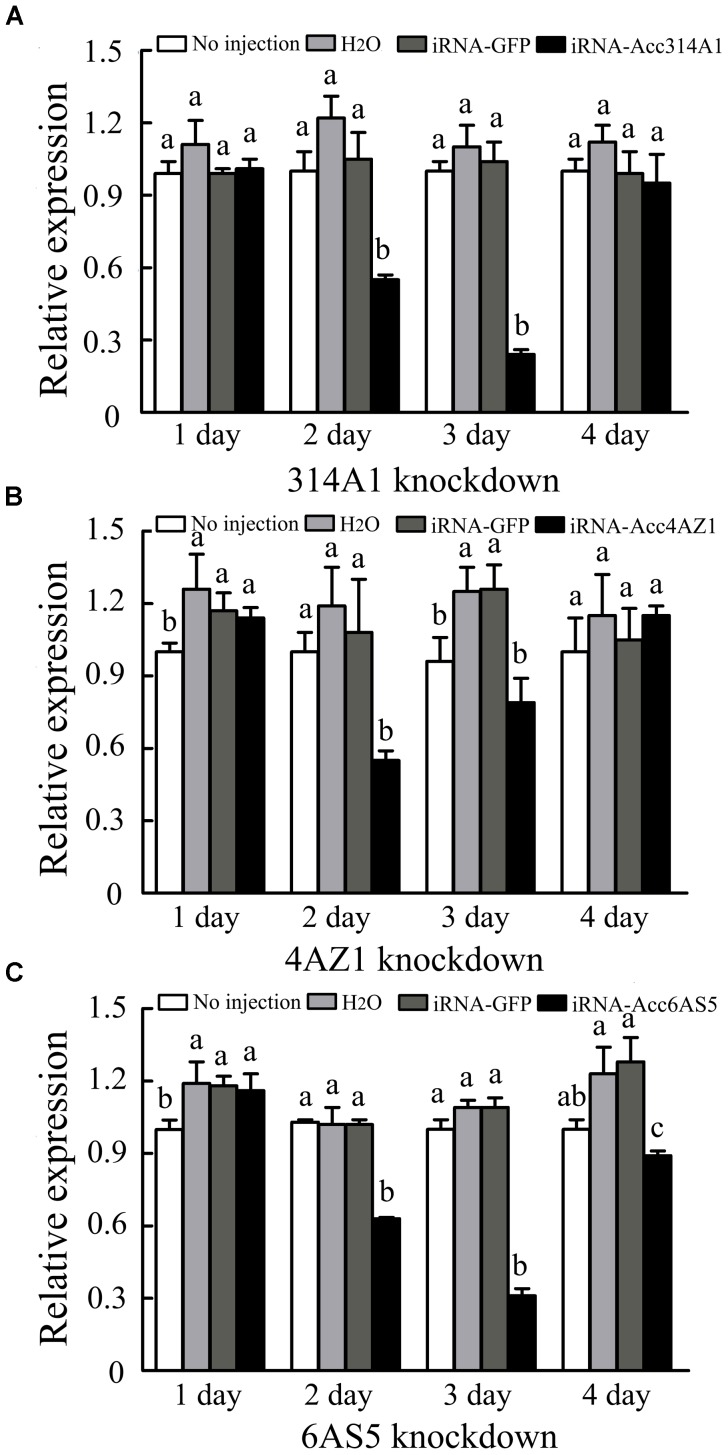
Effects of RNAi on mRNA levels of newly emergence honeybees, as induced by injecting 9 μg dsRNAs. The mRNA levels of **(A)** *AccCYP314A1*, **(B)** *AccCYP4AZ1*, and **(C)** *AccCYP6AS5* are shown. The *β-actin* gene was used as an internal control. Each value is given as the mean + SEM. Different letters above the bars indicate significant differences (*P* < 0.05).

**FIGURE 6:**
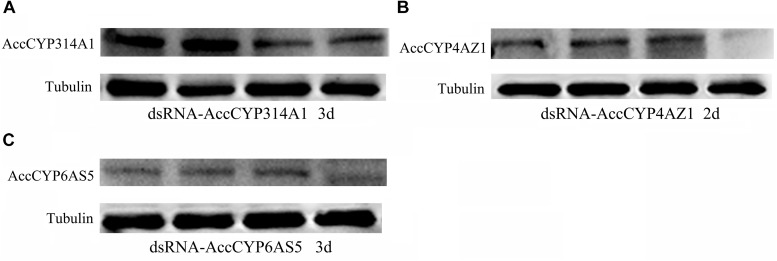
Western blot analysis of AccCYP314A1, AccCYP4AZ1, and AccCYP6AS5 changes after **(A)** *AccCYP314A1*, **(B)** *AccCYP4AZ1*, and **(C)** *AccCYP6AS5* were silenced.

To investigate the functions of *AccCYP314A1, AccCYP4AZ1*, and *AccCYP6AS5* in the antioxidant defense of honeybee workers, RNAi experiments were performed to ascertain their functions. Newly emerged adult workers were injected with H_2_O, dsRNA-*GFP*, dsRNA-*AccCYP314A1*, dsRNA-*AccCYP4AZ1*, or dsRNA-*AccCYP6AS5*. The transcripts of the control groups, the H_2_O and dsRNA-*GFP* groups, were almost equal, showing that injection with dsRNA-*GFP* did not influence the expression of the above three genes in *A. c. cerana* (Figure 5). qPCR results showed that the *AccCYP314A1, AccCYP4AZ1*, and *AccCYP6AS5* genes were successfully silenced compared with the control groups, and the lowest transcript levels were discovered at 2 or 3 days post-injection. Additionally, the Western blot results showed the protein expression levels of the three P450 genes at 2 or 3 days post-injection (Figure 6). As shown in Figures 6A,C, 3 days after injection, the protein expression level of AccCYP314A1 and AccCYP6AS5 were obviously silenced; the protein expression level of AccCYP4AZ1 was downregulated 2 days after injection (Figure 6B). The protein expression levels of AccCYP314A1, AccCYP4AZ1, and AccCYP6AS5 after injection are consistent with the qPCR results.

**FIGURE 7:**
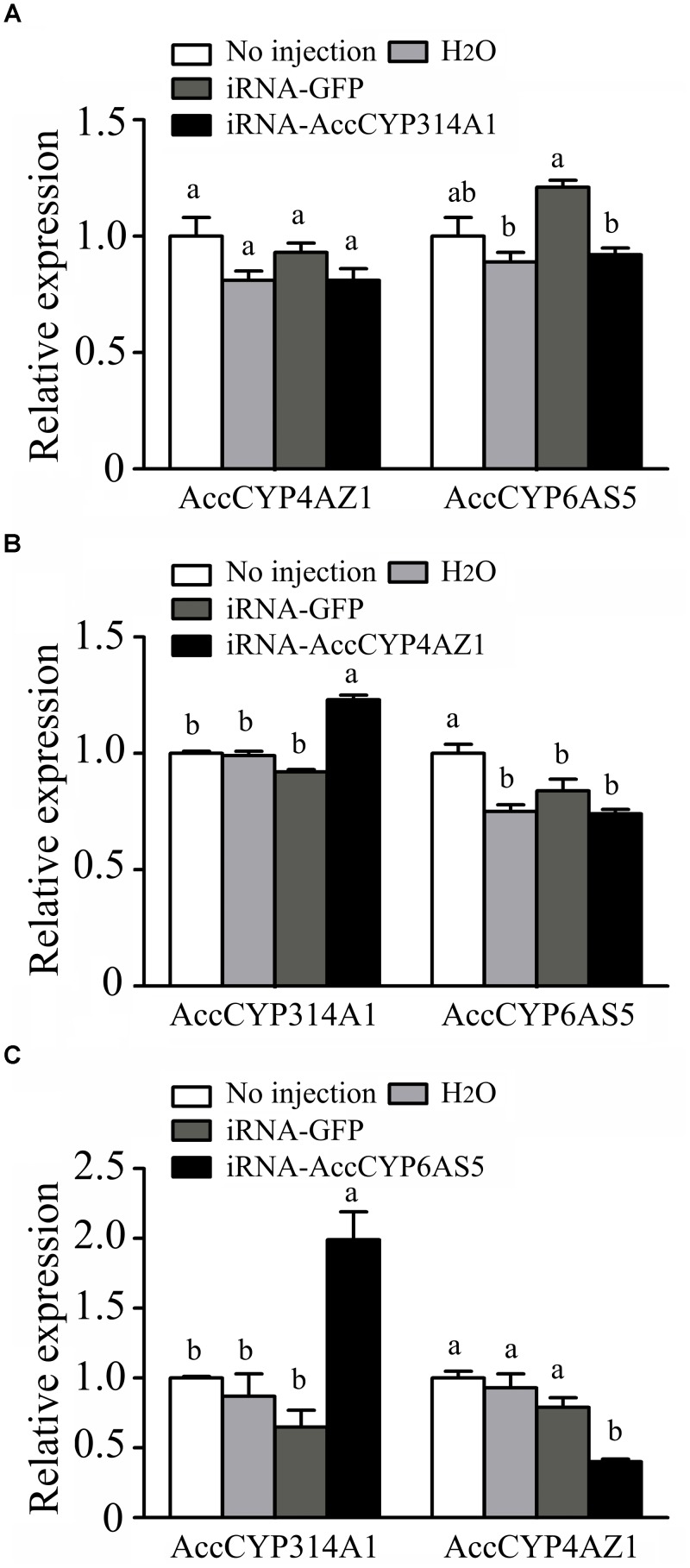
Transcription levels of other two genes performed using qPCR when **(A)** *AccCYP314A1*, **(B)** *AccCYP4AZ1*, and **(C)** *AccCYP6AS5* were knocked down.

To evaluate the response of the other two P450 genes after one was silenced, qPCR was used. As shown in Figure 7A, when *AccCYP314A1* was silenced, no differences in the transcription levels of *AccCYP4AZ1* and *AccCYP6AS5* were apparent among the treatment groups 3 days after injection. When *AccCYP4AZ1* was silenced, the transcription level of *AccCYP314A1* increased, but the transcription level of *AccCYP6AS5* in the H_2_O-injected group, the GFP-injected group and the dsRNA-AccCYP4AZ1-injected group were all downregulated compared with the uninjected group after 2 days (Figure 7B). When *AccCYP6AS5* was knockdown for 3 days, the expression level of *AccCYP314A1* was upregulated, while the expression level of *AccCYP4AZ* was downregulated (Figure 7C).

**FIGURE 8:**
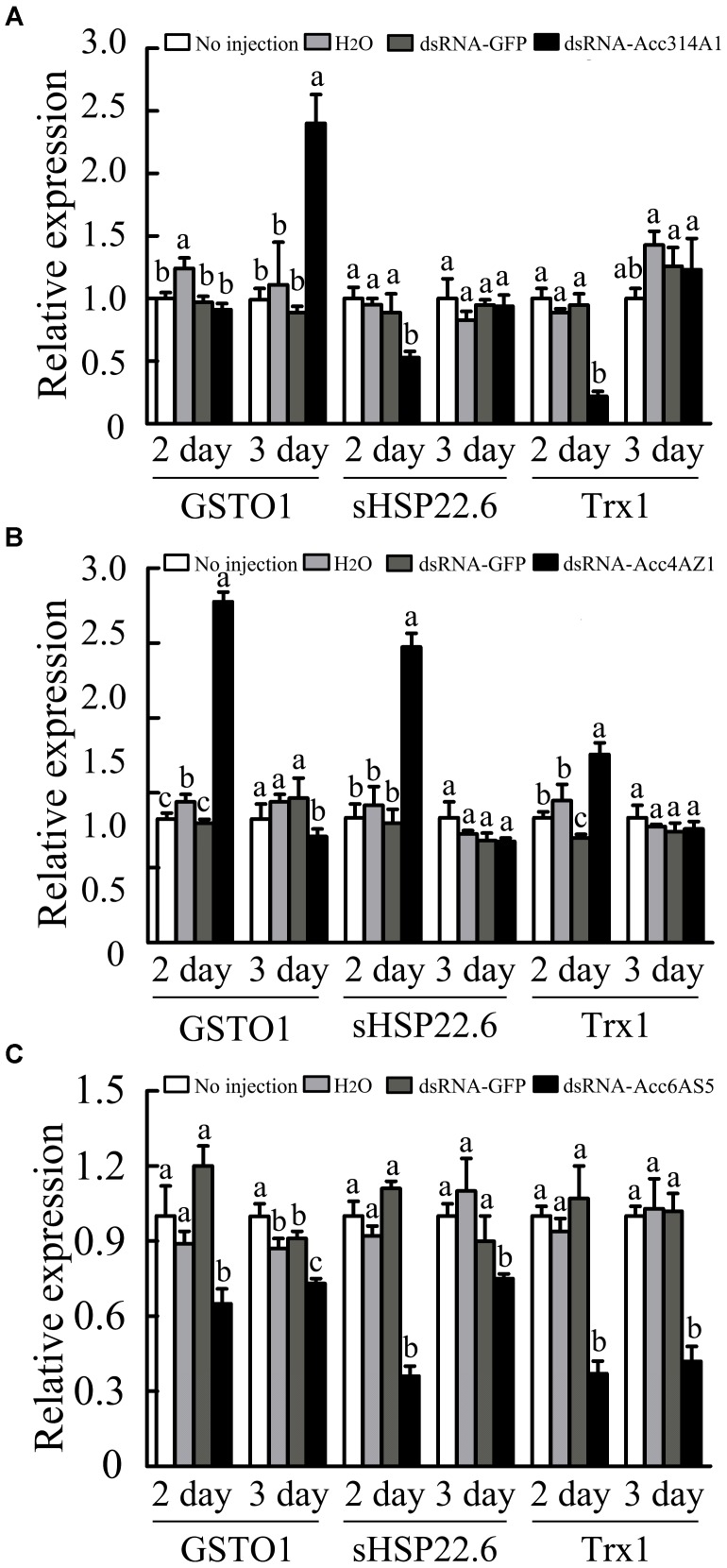
Expression profiles of other antioxidant genes performed using qPCR when **(A)** *AccCYP314A1*, **(B)** *AccCYP4AZ1*, and **(C)** *AccCYP6AS5* were knocked down. The *β-actin* gene was used as an internal control. Each value is given as the mean + SEM. Different letters above the bars indicate significant differences (*P* < 0.05), according to SAS software 9.1.

Quantitative PCR was performed to further evaluate the responses of several genes that have been reported to be involved in oxidative stress responses after *AccCYP314A1, AccCYP4AZ1*, and *AccCP6AS5* were silenced (Meng et al., 2014; Yao et al., 2014; Zhang et al., 2014). When *AccCYP314A1* was knocked down, *AccGSTO1* was induced (Figure 8A). As shown in Figure 8B, *AccGSTO1* and *AccsHsp22.6* were upregulated compared with the control groups. The qPCR results showed that *AccGSTO1, AccsHsp22.6*, and *AccTrx1* were all suppressed when *AccCYP6AS5* was silenced (Figure 8C). All of the above results suggest that the induced genes participate in the compensation for the knockdown of *AccCYP314A1, AccCYP4AZ1*, and *AccCP6AS5* in *A. c. cerana*.

### Determination of Enzymatic Activities After Knockdown of *AccCYP314A1, AccCYP4AZ1*, and *AccCYP6AS5*

**FIGURE 9:**
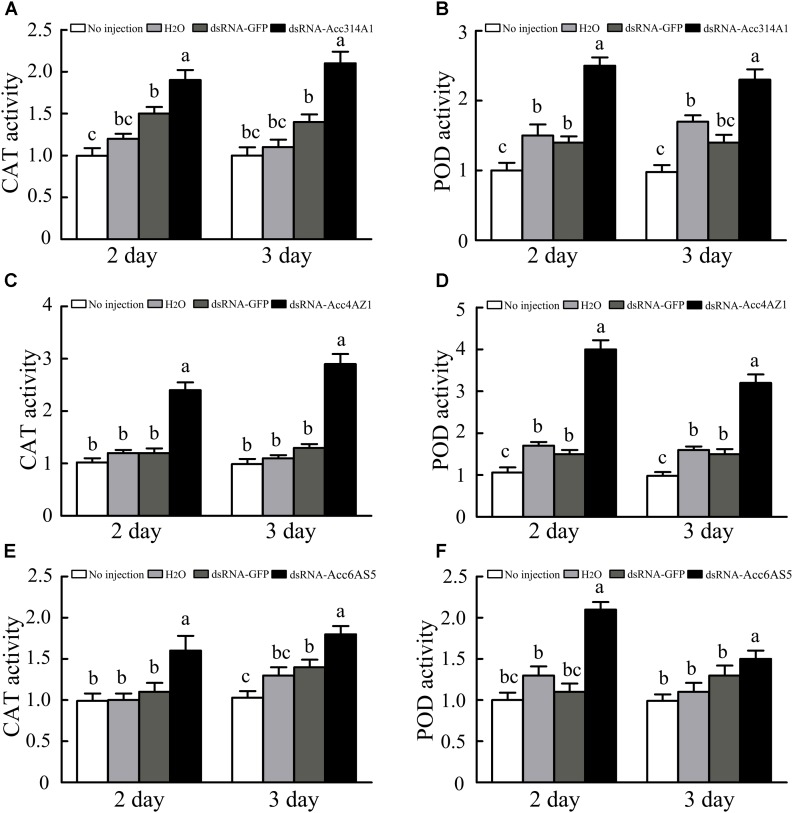
**(A)** Effects of *AccCYP314A1* knockdown on antioxidant enzymatic activities of CAT, day-days after treatment. **(B)** Effects of *AccCYP314A1* knockdown on antioxidant enzymatic activities of POD, day-days after treatment. **(C)** Effects of *AccCYP4AZ1* knockdown on antioxidant enzymatic activities of CAT, day-days after treatment. **(D)** Effects of *AccCYP4AZ1* knockdown on antioxidant enzymatic activities of POD, day-days after treatment. **(E)** Effects of *AccCYP6AS5* knockdown on antioxidant enzymatic activities of CAT, day-days after treatment. **(F)** Effects of *AccCYP6AS5* knockdown on antioxidant enzymatic activities of POD, day-days after treatment. Different letters above the bars indicate significant differences (*P* < 0.05), according to SAS software.

When the *AccCYP314A1, AccCYP4AZ1*, and *AccCYP6AS5* were knock down, the enzymatic activities were all higher compared with control groups, respectively (Figure 9). As shown in Figures 9A,B, the CAT and POD capacities of *A. c. cerana* after the silencing of *AccCYP314A1* were all higher than those of the control groups. Overall, for CAT, the activity increased at 3-day time point significantly higher than *AccCYP4AZ1* was silenced after 2 days (Figure 9C). In *A. c. cerana*, silenced *AccCYP4AZ1* had a significant effect on the activity of POD. The enzymatic activity of POD were significantly higher compared with control bees (Figure 9D). Both CAT and POD activity dose significantly enhanced compared with the control groups when *AccCYP6AS5* was silenced after 2 or 3 days (Figures 9E,F).

### Survival Rate of Artificially Reared Bees

**FIGURE 10:**
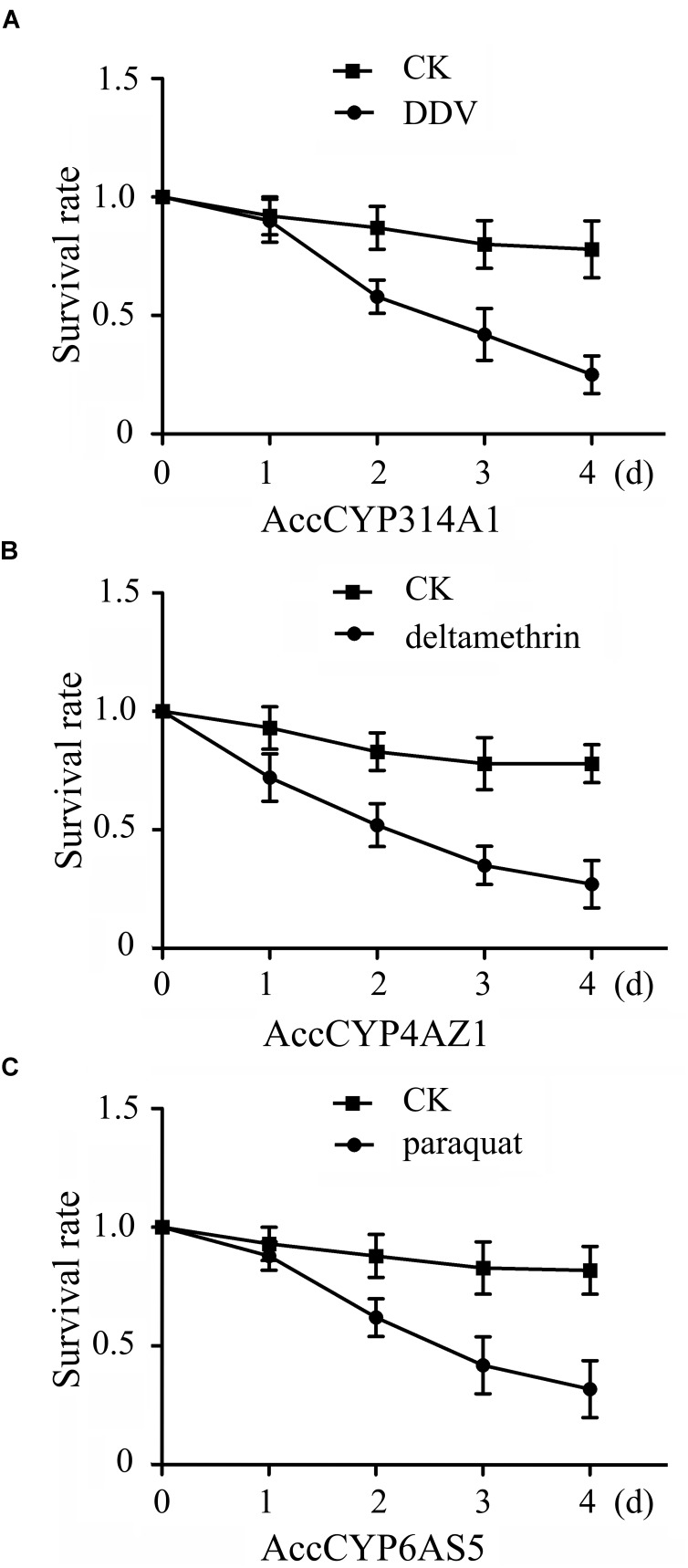
Mean ± SEM (*n* = 3). Survival rate of captive worker bees. **(A)** Effect of DDV on *AccCYP314A1* knockdown workers mortality at different time intervals. **(B)** Effect of deltamethrin on *AccCYP4AZ1* knockdown workers mortality at different time intervals. **(C)** Effect of paraquat on *AccCYP6AS5* knockdown workers mortality at different time intervals. Means capped with different letters are significantly different (Tukey’s HSD, *P* < 0.05).

The three different pesticides treatment groups were all survived shorter compared with control groups, respectively (Figure 10). For *AccCYP314A1*, there was no significant difference between control group and DDV treatment until day 1. By day 2, the control group had significantly higher survival rate compared with the DDV treatment (Figure 10A).

For *AccCYP4AZ1*, there was no significant difference between control group and deltamethrin treatment until day 1. By day 2, honeybees fed the control diet had significantly higher survival rate compared with honeybees fed deltamethrin (Figure 10B).

For *AccCYP6AS5*, honeybees fed the control diet had significantly higher survival rate compared with paraquat treatment starting on day 2, and the trend continued until day 4 (Figure 10C).

## Discussion

In many insects, P450 genes are known to play crucial roles in the metabolism of chemicals from host plants and the degradation of diverse insecticides (pyrethroids, organophosphates, and carbamates), resulting in the bioactivation or detoxification of these compounds (Feyereisen, 1999; Scott, 1999; David et al., 2013). However, only a few P450 genes from *A. c. cerana* have been reported. Therefore, the identification and characterization of new P450 genes have become very attractive research areas. In this study, we obtained the ORF sequences of three new P450 genes (*AccCYP314A1, AccCYP4AZ1*, and *AccCYP6AS5*). P450 genes are classified into four clades based on their amino acid sequence similarities. Within the same clades, P450s share ≥55% identities among amino acid (AA) sequences, whereas individual P450 enzymes can have up to 97% identity with any other P450 enzyme (Nebert et al., 1987). As shown in Figure 1, *AccCYP314A1* and *AccCYP4AZ1* were clustered in the mitochondrial P450 and CYP4 clades, respectively. Phylogenetic analysis showed that AccCYP6AS5 was clustered in the CYP3 clade (Figure 1). The CYP3 and CYP4 clades were once thought to be largely responsible for xenobiotic metabolism and insecticide resistance and are induced by phenobarbital, pesticides, and natural products (Berenbaum, 2002). Meanwhile, the CYP4 clade has been reported to be involved in chemical communication and pesticide metabolism in several insects (Claudianos et al., 2006). Additionally, previous research reported that the CYP4 clade is implicated in the response to oxidative stress and may play crucial roles in protecting *A. c. cerana* from oxidative damage (Shi et al., 2013).

Considering the conserved functional motifs of the P450s, which are present in their protein sequences, almost all of the identified P450 genes are likely to be functional. There are three conserved sequences and functionally important motifs, including WXXXR, EXXR, and FXXGXRXCXG (Supplementary Figures S1–S3). The three motifs are universal among almost all P450 enzymes and are often considered to be a “signature” of P450 enzymes (Feyereisen, 2012). Their sequences are highly diversified, which suggests that each of the identified P450 genes may play a different role or that they may have different substrate specificities (Guo et al., 2016).

Previous research reported that several tissues, including BR, MG, MS, and EP, were involved in pesticide detoxification in several insect species. For example, the predominant expression pattern in the EP suggests that the expression profiles of the three genes are tissue-specific. A plausible explanation for this may be that the EP is the most exposed to external attack and plays a key role in the resistance to abiotic stress (Marionnet et al., 2003). The BR of an insect has been found to detoxify drugs and xenobiotics to protect the insect nervous system (Holmes et al., 2008). In insects, the MG is known to be the most important organ of digestion and absorption and has a defensive function against xenobiotics. Thus, the diversification of expression profiles for P450 genes in the BR, MG, MS, and EP support their potential roles as antioxidants (Figure 2B), although these P450 genes may also play other physiological roles in these tissues.

The relative expression levels of different P450 genes were found to be diversified in different developmental stages of *A. c. cerana*. For example, CYP4G11 was expressed at higher levels in the 14-day post-emergence stage than in other developmental stages, a finding that has been previously reported (Shi et al., 2013). As shown in Figure 2A, *AccCYP314A1*, Acc*CYP4AZ1*, and Acc*CYP6AS5* were all observed to be expressed in all developmental stages. Additionally, *AccCYP314A1*, and *AccCYP4AZ1* were expressed at higher levels of transcript accumulation in Pd, and *AccCYP6AS5* showed significantly higher expression in NE than in the other developmental stages, for which stage *AccCYP314A1* showed the lowest expression (Figure 2A). The above results suggest that the three genes could be involved in specific functions in the Pd and NE stages. Previous studies also found similar results for P450s in *L. migratoria*, where *CYP408B1* was detected at a higher expression level in adults (Guo Y. et al., 2012). However, the expression profiles of these three P450 genes were different from those of *AccCYP336A1* in *A. c. cerana* and *CYP409A1* in *L. migratoria* (Guo Y.Q. et al., 2012; Zhu et al., 2016). In addition, the differences in the expression patterns of the P450 genes during different developmental stages and in different tissues imply their specific functions in insects (Chung et al., 2009).

Previous research findings have revealed that environmental conditions, such as temperature (cold or heat), heavy metals, insecticides, and UV exposure, can induce oxidative stress (Lushchak, 2011; Kottuparambil et al., 2012). Deltamethrin and pyrethroid are still being widely used as major insecticides. In insects, a previous study reported that P450 genes were involved in the process of insecticide resistance. *AccCYP336A1*, a stress-inducible gene, could be induced by various stresses, such as deltamethrin, heat, H_2_O_2_, and UV (Zhu et al., 2016). *CYP409A1* and *CYP408B1* from *L. migratoria* were upregulated after deltamethrin treatment, revealing that *CYP409A1* and *CYP408B1* play key roles in reducing harmful ROS (Guo Y.Q. et al., 2012). Previous research showed that *AccCYP4G11* was upregulated by treatments with 4°C temperatures, H_2_O_2_, several insecticides (cyhalothrin, acaricide, parquet, phoxime) and HgCl_2_ (Shi et al., 2013). Previous results have revealed that some P450 genes (*CYP6FD2, CYP6FF1*, and *CYP6FG2*) showed higher expression levels when treated with the LD10 of deltamethrin but were not induced at the LD30 and LD50 levels of deltamethrin (Guo et al., 2016). These differences could be due to deltamethrin affecting the expression profiles of different P450 genes differently and in a dose-dependent manner. As a previous report described, temperature (heat or cold stress) is one of the key mediators of ROS generation, which could cause physiological changes in organisms (An and Choi, 2010). For example, research has shown that heat stress can induce polyamine oxidation due to ROS damage; cold stress can result in the apoptosis of hepatocytes and liver endothelial cells and the generation of ROS (Rauen et al., 1999). As shown in Figure 3, after treatment with cold (4 °C) or heat (44 °C), the expression levels of *AccCYP314A1, AccCYP4AZ1*, and *AccCYP6AS5* were increased in this study. These results suggest that *AccCYP314A1, AccCYP4AZ1*, and *AccCYP6AS5* may be involved in regulating body temperature and the heat ceiling, thus protecting *A. c. cerana* from ROS damage. It was also demonstrated that heavy metals can damage normal development and enhance the endogenous ROS levels in insects (Narendra et al., 2007). Mercury (Hg) and cadmium (Cd), the most poisonous heavy metals in nature, can directly bind to metal ion sites on enzymes, resulting in the inactivation of enzymes (Rashed, 2001). When foragers forage for pollen and nectar, foragers may come into greater contact with heavy metals in the environment. qPCR results also proved that the expression levels of *AccCYP314A1, AccCYP4AZ1*, and *AccCYP6AS5* were enhanced by CdCl_2_ (Figure 3C) and HgCl_2_ (Figure 3D) treatment. These results suggest that *AccCYP314A1, AccCYP4AZ1*, and *AccCYP6AS5* are involved in avoiding injury under conditions of HgCl_2_ and CdCl_2_ stress. Insecticides, which are the primary threat to honeybees in the environment, could destroy physiological and biochemical functions due to lipid biomembrane oxidation damage (Narendra et al., 2007). In insects, P450 genes have been reported to be involved in insecticide resistance. Several insecticides, such as deltamethrin and pyrethroid, have been widely used and are still being used. In this study, we investigated the effect of three insecticides (deltamethrin, paraquat, and DDV) on *AccCYP314A1, AccCYP4AZ1*, and *AccCYP6AS5* expression at the mRNA and protein levels, to determine whether the three P450 genes are involved in the process of insecticide metabolization. As shown in Figure 3, the expression levels of *AccCYP314A1, AccCYP4AZ1*, and *AccCYP6AS5* were all induced compared with the control samples, although the time to reach peak expression were different for all three (Figures 3E–G). It is a complex manner in which an insecticide affects the expression pattern of different P450 genes differently and which is a time-dependent manner. The differences could be due to the more pronounced toxic effects of pesticides over the long term. UV radiation, a typical oxidant, causes oxidative damage (Schauen et al., 2007; Nguyen et al., 2009). Here, our data showed that *AccCYP314A1, AccCYP4AZ1*, and *AccCYP6AS5* can be induced by UV radiation treatment (Figure 3H). These findings support the hypothesis that *AccCYP314A1, AccCYP4AZ1*, and *AccCYP6AS5* play crucial roles in protecting *A. c. cerana* against ROS damage. The regulation of molecular mechanisms by different P450 genes in honeybees is not well understood. If the gene are involved in the process of insecticide resistance, the expression patterns of P450 genes may affect the susceptibility of insects to insecticides.

Western blot analysis was performed to explore the protein expression patterns of *AccCYP314A1, AccCYP4AZ1*, and *AccCYP6AS5* after *A. c. cerana* were treated with 4 °C, CdCl_2_, paraquat and deltamethrin. As a whole, the protein expression patterns of *AccCYP314A1, AccCYP4AZ1*, and *AccCYP6AS5* were consistent with the qPCR data. Concerning the mRNA and protein levels of *AccCYP314A1, AccCYP4AZ1*, and *AccCYP6AS5*, there were certain differences in time points and degree of change (Figure 4). There are some explanations for these differences: first, the increased protein levels of *AccCYP314A1, AccCYP4AZ1*, and *AccCYP6AS5* could be the result of accumulation; second, the inconsistent mRNA and protein levels could be due to post-transcriptional regulation. Indeed, Hfq (RNA-binding protein) was shown to regulate the expression of invE through post-transcriptional regulation in *S. sonnei*; although the mRNA expression of *invE* was easy to detect, the protein of invE was barely detected (Mitobe et al., 2009). The above results support the hypothesis that *AccCYP314A1, AccCYP4AZ1*, and *AccCYP6AS5* play important roles in protecting *A. c. cerana* against ROS damage.

To further understand gene roles, RNAi technology has been used in many insects. To examine the potential roles of these three genes, we first established gene-silencing procedures, in which H_2_O, dsRNA-*GFP*, dsRNA-*AccCYP314A1*, dsRNA-*AccCYP4AZ1*, or dsRNA-*AccCYP6AS5* was microinjected into *A. c. cerana*. The controllable dose and minimal invasiveness make the microinjection method widely useful for diverse insect types. The results showed that the effect of dsGFP was less obvious; there were no off-target effects because there is no GFP target in *A. c. cerana* (Elias-Neto et al., 2010). As shown in Figures 5, 6, the gene silencing efficiency was different for the three genes. In this study, 40–70% gene silencing efficiency was achieved for the different genes. Above results showed that the three P450 genes of *AccCYP314A1, AccCYP4AZ1*, and *AccCYP6AS5* might be involved in environmental response and detoxification in insects. The qPCR method was performed to elucidate the relationship among the three P450 genes. As shown in Figure 7, the transcription levels of the other two genes were significant changed compared with control groups when one of the three genes was silenced. These results should be useful for understanding the relationship and the functions of the three P450 genes.

The RNAi technology provides a valuable research tool for future research into the functions of P450s in *A. c. cerana*. Compared with the control groups, *AccCYP314A1* silencing markedly down-regulated *AccsHSP22.6* and *AccTrx1* on the second day post injection, but *AccGSTO1* expression was up-regulated on the third day post injection, suggesting that *AccGSTO1* may be involved in the process of reducing the production of ROS after *AccCYP314A1* is silenced (Figure 8A). The qPCR results also showed that, when *AccCYP4AZ1* was silenced, the expression levels of *AccGSTO1, AccTrx1*, and *AccsHSP22.6* were induced (Figure 8B). In addition, when *AccCYP6AS5* was knocked down, the expression levels of *AccGSTO1, AccTrx1*, and *AccsHSP22.6* were lower than those of the control groups (Figure 8C). Among these genes, *AccGSTO1* and *AccTrx1* were demonstrated to be involved in different abiotic stress responses (Meng et al., 2014; Yao et al., 2014). Previous research showed that *AccsHSP22.6* could not only been involved in the abiotic stress response but also in development (Zhang et al., 2014). Thus, we speculate that *AccCYP314A1* and *AccCYP6AS5* might also be involved in the process of abiotic stress defense and development in *A. c. cerana*.

The antioxidant enzymes CAT and POD play crucial roles in scavenging ROS, which can cause oxidative damage to DNA, proteins, and lipids in an organism (Lee et al., 2005; Corona and Robinson, 2006). As shown in our results, the activities of CAT and POD were all enhanced when *AccCYP314A1, AccCYP4AZ1*, and *AccCYP6AS5* were knocked down, suggesting that *A. c. cerana* was exposed to a high level of oxidative stress when *AccCYP314A1, AccCYP4AZ1*, and *AccCYP6AS5* were silenced, and the two antioxidant enzymes may be involved in scavenging ROS (Figures 9A–C). When *AccCYP314A1, AccCYP4AZ1*, and *AccCYP6AS5* were silenced, the DDV, deltamethyrin, and paraquat treatments significantly decreased survival rate, respectively (Figure 10). This results further confirmed that enzymes encoded by the three P450 genes might contribute to the detoxification of the three pesticides in *A. c. cerana* (Figure 10).

In this study, we identified and characterized the expression patterns of three novel P450 genes from the major agricultural insect *A. c. cerana*. The expression patterns of the three novel genes showed developmental stage-specific and tissue-specific expression in *A. c. cerana*. Our findings on the three antioxidant genes in *A. c. cerana* reveal that there may be more extensive diversification within the P450 supergene family in insects (Sasabe et al., 2004). Our results also imply that the three genes were involved in antioxidant activities, based on the results of using RNAi knockdown for each of the three genes in *A. c. cerana*. The knowledge gained from this study may help us to better understand the roles of insect P450s and their interactions with pesticides at the mRNA and protein levels. Thus, further functional research on these P450s is essential for the assessment of their functions in abiotic stress and in agriculturally important insects.

## Author Contributions

WZ carried out the experimental work and wrote the paper. BX designed the experiments. WC participated in the SDS-PAGE and Western blot work. ZL designed the primers and analyzed the data of Western blot. LM analyzed the data of RT-qPCR. JY carried out the breeding of *Apis cerana cerana*. HW assisted with transcript expression assessments. ZL carried out RNA extraction and cDNA synthesis. All authors read and approved the final manuscript.

## Conflict of Interest Statement

The authors declare that the research was conducted in the absence of any commercial or financial relationships that could be construed as a potential conflict of interest.

## Footnotes

1 http://www.ncbi.nlm.nih.gov/Structure/cdd/cdd.shtml

## Supplementary Material

FIGURE S1 The alignment of the deduced AccCYP314A1 amino acid sequence with other species known *AccCYP314A1*. Characteristic regions are underlined including Helix-C (WXXXR), Helix-K (EXXR), and the consensus sequence FXXGXRXCXG are marked with two triangle ().

FIGURE S2 The alignment of the deduced AccCYP4AZ1 amino acid sequence with other species known *AccCYP4AZ1*. Characteristic regions are underlined including Helix-C (WXXXR), Helix-K (EXXR), and the consensus sequence FXXGXRXCXG are marked with two triangle ().

FIGURE S3 The alignment of the deduced AccCYP6AS5 amino acid sequence with other species known *AccCYP6AS5*5. Characteristic regions are underlined including Helix-C (WXXXR), Helix-K (EXXR), and the consensus sequence FXXGXRXCXG are marked with two triangle ().

FIGURE S4 The nucleotide sequences multiple alignment among the three target genes.

FIGURE S5 The specific antibodies against the three target proteins. Protein marker molecular weights are given in kDa. **(A)** the specific antibodies anti-AccCYP314A1 against AccCYP314A1; M, protein marker; lines 1–4: the bands of AccCYP314A1. **(B)** The specific antibodies anti-AccCYP4AZ1 against AccCYP4AZ1; M, protein marker; lines 1–4: the bands of AccCYP4AZ1. **(C)** The specific antibodies anti-AccCYP6AS5 against AccCYP6AS5; M, protein marker; line 1; the negative sample (only SDS-PAGE loading buffer and RIPA buffer); lines 2–4: the bands of AccCYP6AS51.

TABLE S1 Abiotic stress conditions for each experimental group.
