# Supplementary figures and images for: Identification and Characterization of Three New Cytochrome P450 Genes and the Use of RNA Interference to Evaluate Their Roles in Antioxidant Defense in Apis cerana cerana Fabricius
Source: Front Physiol. 2018 Nov 15;9:1608. doi: 10.3389/fphys.2018.01608 (PMC6250095; doi:10.3389/fphys.2018.01608)

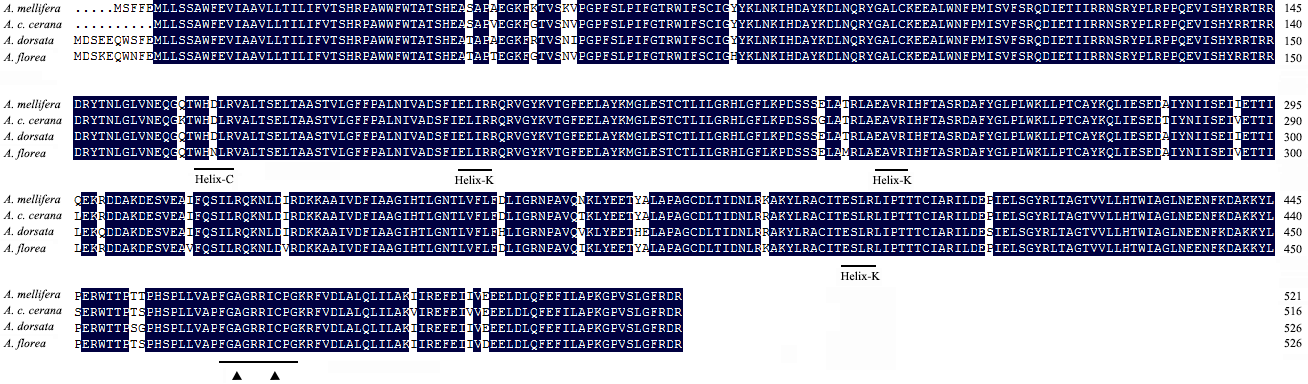

Supplement: FIGURE S1 — The alignment of the deduced AccCYP314A1 amino acid sequence with other species known AccCYP314A1. Characteristic regions are underlined including Helix-C (WXXXR), Helix-K (EXXR), and the consensus sequence FXXGXRXCXG are marked with two triangle (). [file Image_1.TIF]

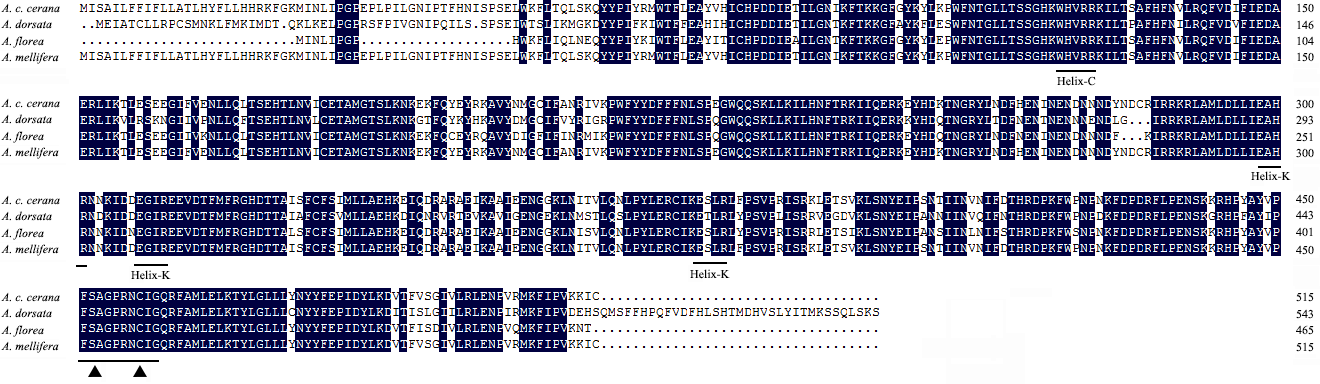

Supplement: FIGURE S2 — The alignment of the deduced AccCYP4AZ1 amino acid sequence with other species known AccCYP4AZ1. Characteristic regions are underlined including Helix-C (WXXXR), Helix-K (EXXR), and the consensus sequence FXXGXRXCXG are marked with two triangle (). [file Image_2.TIF]

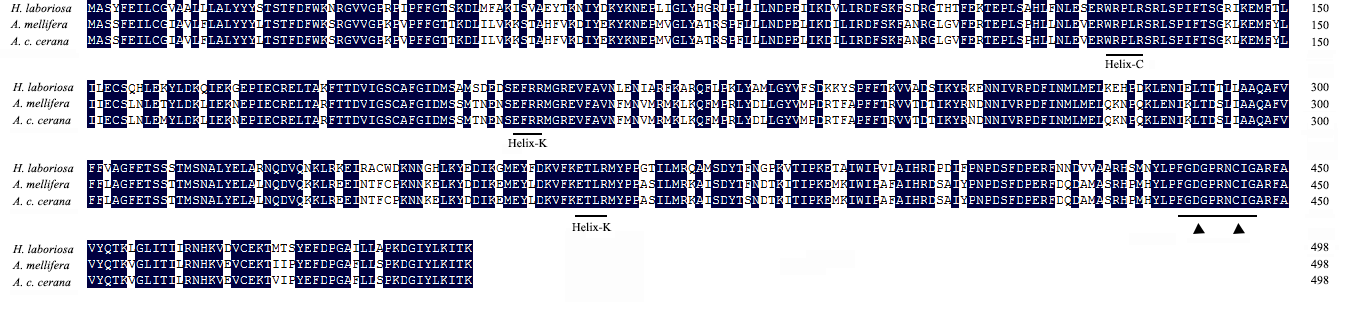

Supplement: FIGURE S3 — The alignment of the deduced AccCYP6AS5 amino acid sequence with other species known AccCYP6AS55. Characteristic regions are underlined including Helix-C (WXXXR), Helix-K (EXXR), and the consensus sequence FXXGXRXCXG are marked with two triangle (). [file Image_3.TIF]

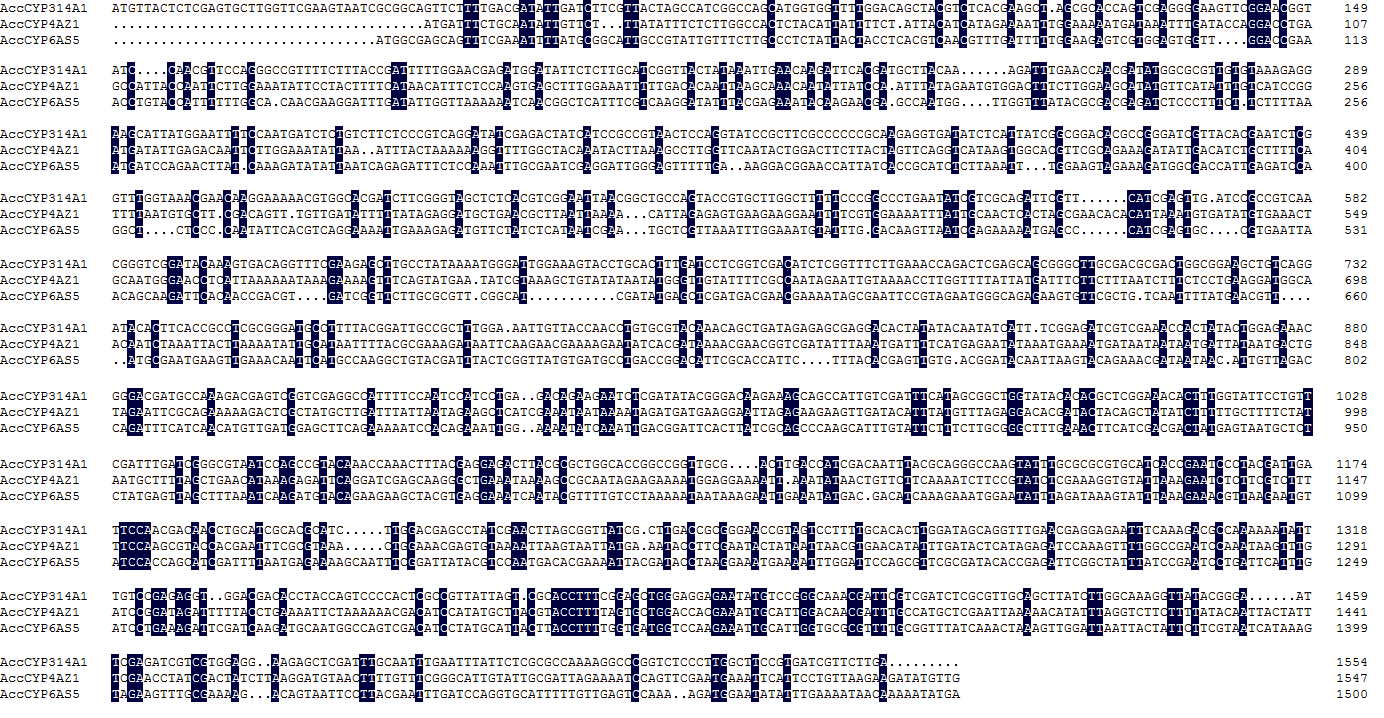

Supplement: FIGURE S4 — The nucleotide sequences multiple alignment among the three target genes. [file Image_4.TIF]

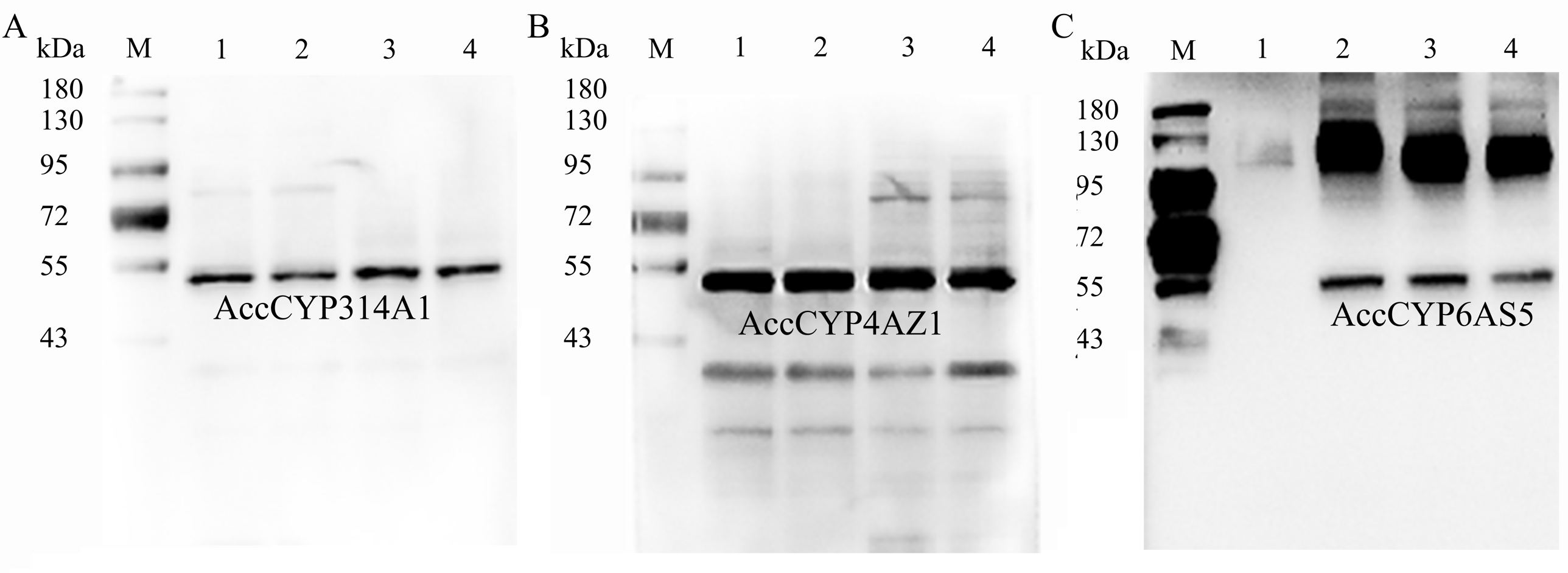

Supplement: FIGURE S5 — The specific antibodies against the three target proteins. Protein marker molecular weights are given in kDa. (A) the specific antibodies anti-AccCYP314A1 against AccCYP314A1; M, protein marker; lines 1–4: the bands of AccCYP314A1. (B) The specific antibodies anti-AccCYP4AZ1 against AccCYP4AZ1; M, protein marker; lines 1–4: the bands of AccCYP4AZ1. (C) The specific antibodies anti-AccCYP6AS5 against AccCYP6AS5; M, protein marker; line 1; the negative sample (only SDS-PAGE loading buffer and RIPA buffer); lines 2–4: the bands of AccCYP6AS51. [file Image_5.TIF]
