# Supplementary material for: Identification and Characterization of Three New Cytochrome P450 Genes and the Use of RNA Interference to Evaluate Their Roles in Antioxidant Defense in Apis cerana cerana Fabricius
Source: Front Physiol. 2018 Nov 15;9:1608. doi: 10.3389/fphys.2018.01608 (PMC6250095; doi:10.3389/fphys.2018.01608)
Supplement: TABLE S1 — Abiotic stress conditions for each experimental group. [file Table_1.DOC]

**Supplementary Table 1.** Abiotic stress conditions for each experimental group.

| Experiment condition | producer and purity | Treatment method | Collection time  after treatment |
| --- | --- | --- | --- |
| 4 °C |  | Exposure | 0, 0.25, 0.5, 1, 2, 3 and 5 h |
| 44 °C |  | Exposure | 0, 0.25, 0.5, 1, 2, 3 and 5 h |
| CdCl_2_ (2 mg/mL) | Xiya, Shandong, China; 99.7% | Feed | 0, 1.5, 3, 4.5, 6, 12 and 24 h |
| HgCl_2_ (3 mg/mL) | Kaitong, Tianjing, China; 99.7% | Feed | 0, 1.5, 3, 4.5, 6, 12 and 24 h |
| Deltamethrin (25 mg/L) | Alta Scientific Co., Ltd.; 98%+ | Feed | 0, 1, 2, 3, 4, 5 and 6 h |
| Paraquat (25 mg/L) | Alta Scientific Co., Ltd.; 98%+ | Feed | 0, 1, 2, 3, 4, 5 and 6 h |
| DDV (0.8 g/L) | Alta Scientific Co., Ltd.; 98%+ | Feed | 0, 1, 2, 3, 4, 5 and 6 h |
| UV (30 mJ/cm^2^) |  | Exposure | 0, 1, 2, 3, 4, 5 and 6 h |
